# 

**Published:** 1846-11

**Authors:** 


### WESTERN JOURNAL

OF

### MEDICINE AND SURGERY.

EDITORS.

---

DANIEL DRAKE, M.D.,

PROFESSOR OF PATHOLOGY AND THE PRACTICE OF MEDICINE
IN THE MEDICAL INSTITUTE OF LOUISVILLE:

LUNSFORD P. YANDELL, M.D.,

PROFESSOR OF CHEMISTRY AND PHARMACY IN THE SAME:

THOMAS W. COLESCOTT, M. D.

### RECEIPTS OF MEDICAL JOURNAL FOR OCTOBER, 1846.

| | |
|---|---|
| Dr. J. Culbertson, Vicksburgh, Miss., | $7 50 |
| J. R. Moore, Jefferson County, Ky., | 5 00 |
| F. A. Ramsey, Memphis, Tenn., | 5 00 |
| W. Dulaney, Brountville, Tenn., | 5 00 |
| —— Murray, White River, Ark., | 5 00 |

The Western Journal of Medicine and Surgery is published monthly by the undersigned, at the corner of Main and Fifth streets, Louisville, at $5 per annum, payable in advance. Each number contains 96 pages, making two volumes in the year of more than 550 pages.

Contributions to its pages by the Physicians of the Valley of the Mississippi, addressed to the Editors, are respectfully solicited.

Letters on business, to be addressed, postage paid, to the publishers.

*January*, 1844. PRENTICE & WEISSINGER.

